# Segmentectomy is associated with worse disease-free survival compared to lobectomy in patients with stage IA3 non-small cell lung cancer

**DOI:** 10.1007/s00423-025-03835-0

**Published:** 2025-08-15

**Authors:** Kaman Hafsa, Stork Theresa, Okumus Özlem, Alnajdawi Yazan, Jemsi Mehran, Petrone Ana, Collaud Stéphane, Stéphane Collaud

**Affiliations:** Department of Thoracic Surgery, Cologne-Merheim Hospital, University of Witten/Herdecke, Cologne, Germany

**Keywords:** Lung cancer, Stage IA3, Segmentectomy, Lobectomy

## Abstract

**Purpose:**

Segmentectomy has become the new standard of care for selected patients with stage IA1-2 non-small cell lung cancer (NSCLC). For stage IA3 NSCLC, lobectomy is indicated. This study aims to compare the outcome after segmentectomy and lobectomy in patients with stage IA3 NSCLC.

**Methods:**

We retrospectively reviewed all patients undergoing surgery for NSCLC in our center between 2013 and 2023. We identified all patients who underwent segmentectomy or lobectomy for pathological stage IA3 tumors. Survival was calculated from the date of surgery until last follow- up. Univariate analysis was performed to study the impact on overall survival (OS) and disease-free survival (DFS) of clinical variables.

**Results:**

We identified fifty-nine patients undergoing surgery for stage IA3 NSCLC. Twenty- seven (28%) patients underwent segmentectomy and sixty-eight (72%) patients underwent lobectomy. Median age was 68 years (47–85). Median FEV1 was 74% (39–140), median DLCO was 67% (28–128). Surgery was performed by VATS in most cases (91%). There was no difference in age and lung function between the lobectomy and segmentectomy groups. Five-year OS was 67%. 5-year DFS was 63%. Five-year DFS was significantly worse in patients who underwent segmentectomy compared to lobectomy (37% vs. 72%, *p* = 0.040). There was no difference in 5-year OS (56% vs. 69%, *p* = 0.56).

**Conclusion:**

Segmentectomy is associated with significantly worse disease-free survival compared to lobectomy in our cohort of patients with stage IA3 NSCLC.

## Introduction

NSCLC (non-small cell lung cancer) is the most common form of lung cancer [1]. Patients with NSCLC are often diagnosed at an advanced stage. Without lung cancer screening, only 27% of cases are diagnosed in the early stages I or II [2]. For stage IA NSCLC surgical resection is the treatment of choice [3].Based on the randomized study of The Lung Cancer Study Group, lobectomy has been standard of care for patients in Stage I NSCLC [4].

Two recent large prospective randomized studies have compared the outcome of sublobar resection and lobectomy, favoring sublobar resections for node-negative peripherally located NSCLC ≤ 2 cm. The JCOG0802/WJOG4607L Trial could show that segmentectomy offers significantly better overall survival (OS) and similar disease-free survival (DFS), despite an increased risk of locoregional relapses [5]. The findings of the CALGB 140,503 Trial confirmed the non-inferiority of sublobar resection with respect to disease-free survival, with similar OS compared to lobectomy [6]. Based on these results segmentectomy is now the standard of care in selected patients with node-negative NSCLC ≤ 2 cm (stage IA1-2).

Broadening the indication of segmentectomy for patients with node-negative NSCLC of 2–3 cm (stage IA3) remains controversial. A single arm phase III trial (JCOG1211) showed promising results, demonstrating that segmentectomy for ground glass dominant NSCLC between 2 and 3 cm in size achieved a high 5- years relapse-free survival of 98% [7]. Nonetheless only few studies have attempted to compare the outcome between segmentectomy and lobectomy in this stage [8–11]. To date there is only one ongoing randomized prospective study in Japan WJOG16923L (STEP UP Trial) comparing segmentectomy to lobectomy as a treatment for pure solid NSCLC in stage IA3. This study was initiated in January 2024 and will last 5 years [12]. Here, we conducted a retrospective study to compare the outcome of segmentectomy and lobectomy in patients with stage IA3 NSCLC.

## Material and method

### Study design and population

This study was approved by the ethics committee of the University of Witten/Herdecke (approval number S-178/2024). Informed consent was waived due to the retrospective nature of the study.

All patients with pathological stage I NSCLC who underwent surgery at our center between January 2013 and December 2023 were reviewed. Restaging according to the 8th edition of the TNM classification [13] was performed to identify all patients with pathologic stage IA3. Data were obtained from electronic patient’s chart and retrospectively analyzed. We excluded wedge resections, trisegmentectomies (left upper trisegmentectomies, basal segment resections and other atypical trisegmentectomies), lingula resections as well as resection with dissection of fewer than three mediastinal lymph node stations or fewer than twelve lymph nodes.

All patients were clinically evaluated and were submitted to lung function test. Preoperative assessment relied primarily on chest computed tomography (CT). Additional pre- or postoperative imaging included 18-fluoro-2-deoxyglucose positron emission tomography/computed tomography (FDG-PET/CT), whole-body magnetic resonance imaging (WB-MRI), cerebral MRI (c-MRI), or cerebral CT (c-CT). From October 2022, staging was conducted in accordance with the European Society of Thoracic Surgeons (ESTS) guidelines [14].

### Surgical technique

A video-assisted thoracoscopic approach was favored over thoracotomy. Uniportal video-assisted resections were exclusively performed from October 2022. Preoperative 3-dimensional (3D) reconstruction based on CT for patients eligible for segmentectomy was inconsistently performed. Three-dimension reconstruction was used to define the extent of resection. A lobectomy was planned when the virtual safety margin was < 1 cm or when the margin to tumor ratio (MTR) was < 1. In case of the lack of preoperative diagnosis, lung wedge resection with intraoperative fresh frozen sections were performed before segmentectomy or lobectomy whenever anatomically feasible. Systematic lymphadenectomy was routinely performed according to ESTS guidelines [15]. Intraoperative frozen sections to assess lymph nodes or resections margins were performed at surgeon’s discretion.

### Follow up

All cases were discussed in an interdisciplinary tumor board. Follow-up was advised and conducted with clinical examination and Chest-CT according to German national guidelines [16]. Locoregional recurrence was defined as recurrence within the ipsilateral lung field or in ipsilateral or contralateral mediastinal or hilar lymph nodes. Distant recurrences were defined as recurrences involving the contralateral lung or pleura (ipsilateral or contralateral), or outside of the thoracic cavity. OS was estimated from the date of surgery until death or the most recent follow-up. DFS was measured as the time interval from surgery to the occurrence of tumor recurrence or death of any cause.

### Statistical analysis

The extracted data included age, sex, comorbidities, Eastern Cooperative Oncology Group Performance Status scale (ECOG), lung function and postoperative complications. Tumor characteristics included consolidation to tumor ratio (CTR), histological type, grade, clinical and pathologic T, N and M status, with the number of examined lymph nodes, lymphovascular invasion, status of surgical margins. Continuous variables were presented as median with interquartile ranges and compared between groups using Mann-Whitney U-Test and Student-t-Test. Categorical variables were summarized as frequencies and percentages, with comparison performed using the Pearson chi-square test or Fischer’s exact test where appropriate. OS and DFS were analyzed using the Kaplan-Meier method and compared with the log-rank test. Univariate analysis was conducted to evaluate the effect of clinical and pathological variables on DFS and OS. Statistical significance was set at *p* < 0,05. All analyses were performed using IBM SPSS software (IBM Corp., Armonk, NY, USA).

## Results

Between January 2013 and December 2023, 699 patients with stage I NSCLC underwent surgery. From those, 95 patients had pathological stage IA3. Twenty-seven patients (28%) underwent segmentectomy and sixty-eight patients (72%) underwent lobectomy. Patient and tumor characteristics are summarized in Table 1.

**Table 1:** Patient and tumor characteristics for both lobectomy and segmentectomy groups

Covariate	Lobectomy (*n* = 68)	Segmentectomy (*n* = 27)	*P*-value
Age (mean+/- SD)	67 ($\:\pm\:$8.85)	69 ($\:\pm\:$8.99)	0.44
Gender (n) Male	35 (52%)	12 (44%)	0.53
ECOG (n) 0 1 2	29 (44%) 35 (53%) 2 (3%)	18 (69%) 8 (31%) 0	0.07
Comorbidity (cardiovascular, pulmonary, diabetes, oncological) (n) No Yes	34 (51%) 33 (49%)	14 (52%) 13 (48%)	0.92
Smoking history (n) Never smoker Ex smoker Smoker	6 (9%) 33 (48%) 28 (41%)	4 (15%) 11 (41%) 12 (44%)	0.71
FEV1 (%) (mean +/SD)	77 ($\:\pm\:$18)	72 ($\:\pm\:18$)	0.30
TLCO (%) (mean)	67 ($\:\pm\:$19)	68 ($\:\pm\:$14)	0.89
Surgical approach (n) VATS Thoracotomy Conversion	61 (91%) 5 (8%) 1 (1%)	25 (93%) 1 (4%) 1 (3%)	0.64
Complications (n) No Yes	49(78%) 14 (22%)	24 (89%) 3 (11%)	0.21
CTR (mean)	0.92 ($\:\pm\:0$0.18)	0.94 ($\:\pm\:0.17$)	0.72
Histology (n) Adenocarcinoma Squamous cell carcinoma Other	40 (59%) 16 (23%) 12 (18%)	17 (63%) 8 (30%) 2 (7%)	0.42

*SD *standard deviation, *ECOG* Eastern Cooperative Oncology Group Performance Status scale, *FEV1* forced expiratory volume in 1 second, *DLCO* diffusion capacity of lungs for carbon monoxide, *CTR* Consolidation to tumor ratio

Baseline characteristics did not significantly differ between the two groups. While cardiovascular, pulmonary comorbidities were similarly distributed, other comorbidities including diabetes, thyroid disorders, immunological diseases and prior oncologic conditions were significantly more frequent in the lobectomy group (*p* = 0.002).

Surgery was performed by VATS in most cases (91%). The type and distribution of resected segments are shown in Figure (fig.) 1.

**Fig. 1:**
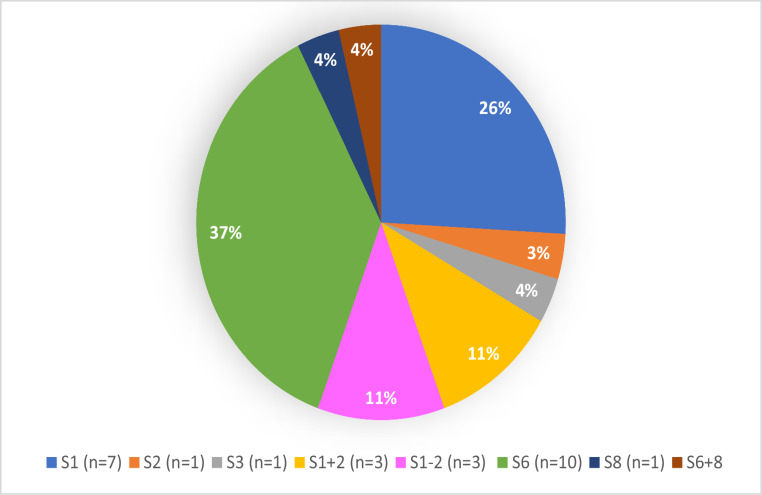
Pie chart illustrating the distribution in percentage of resected segments, S represents Segment

 Postoperative complications occurred in 17 patients (18%). Most common complication was pneumonia (5%), followed by prolonged (more than 5 days) air leak, (4%).

**Fig. 2:**
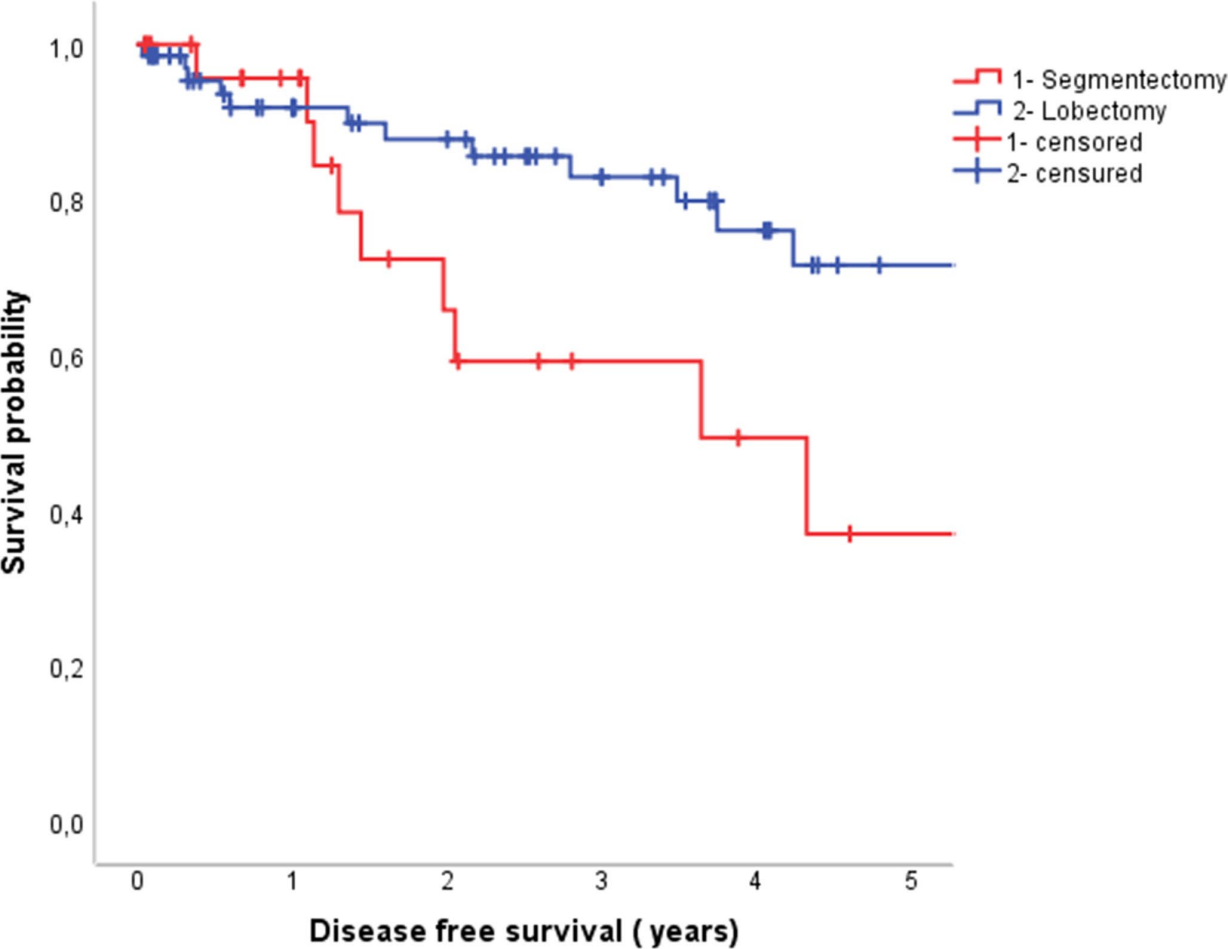
Disease-free survival between the segmentectomy and lobectomy groups

Tumors were mostly purely solid (CTR = 1) in both groups (92% in segmentectomy group vs. 85% in lobectomy group). Patients who underwent lobectomy had significantly larger tumors compared to the segmentectomy group (2.6 cm vs. 2.4 cm, *p* = 0.02). There were more resected lymph nodes in the lobectomy group vs. the segmentectomy group (mean 25 (± 10) and mean 19 (± 8), *p* = 0.007). Mean resection margin was significantly smaller in the segmentectomy group compared to lobectomy group (2.0 (± 1.99) vs. 3,3 (± 1.95), *p* = 0.002). When categorized, the proportion of patients with MTR ≥ 1 was significantly higher in the lobectomy group in comparison to segmentectomy group (61% vs. 27%, *p* = 0.004). Microscopic incomplete R1 resection occurred exclusively in the segmentectomy group in one case. At a median follow-up of 34 months recurrence or death occurred in 9 patients (33%) in the segmentectomy and 13 patients (19%) in the lobectomy group. In the segmentectomy group, 50% of recurrences were locoregional and 50% were distant. In the lobectomy group, 20% of recurrences were locoregional, whereas 80% were distant. Recurrence patterns, whether locoregional or distant, did not significantly differ between segmentectomy and lobectomy (*p* = 0.343). DFS was significantly worse in patients who underwent segmentectomy compared to lobectomy, with 5-year DFS of 37% vs. 72%, respectively (*p* = 0.040) (Fig. 2).

**Fig. 3:**
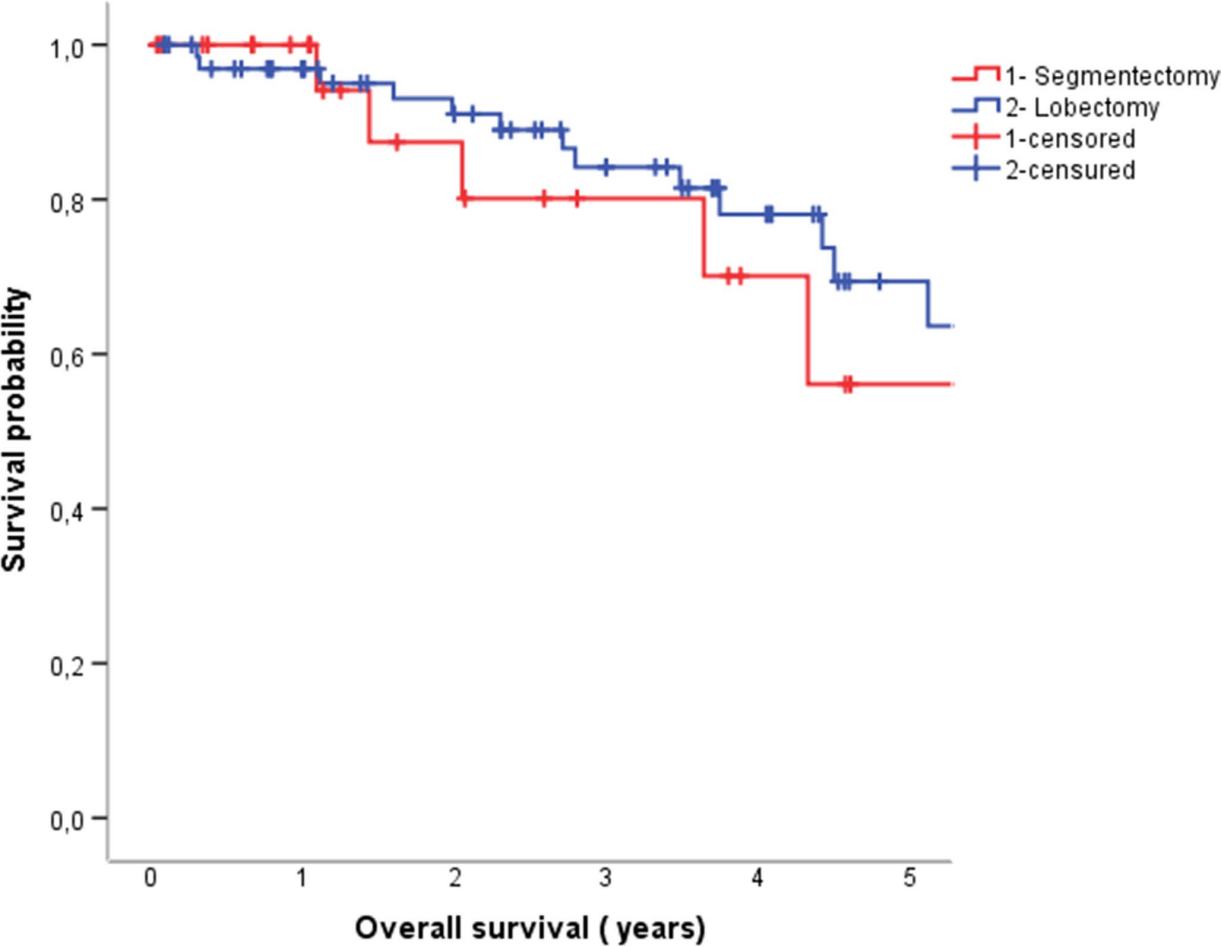
Overall survival between the segmentectomy and lobectomy group

There was no significant difference in 5-year OS 56% vs. 69% respectively (*p* = 0.56) (Fig. 3).

## Discussion

Since the groundbreaking results of the JCOG0802/WJOG4607L and CALGB 140,503 Trial segmentectomy has become the standard of care in selected patients with stage IA1-2 NSCLC [5, 6, 17]. For stage IA3 solid NSCLC, there remains a lack of data comparing the outcome after segmentectomy and lobectomy. In our study we showed that DFS was worse for patients after segmentectomy compared to lobectomy, while there was no difference in OS for both groups.

In a large, retrospective study from the Japanese Joint Committee of Lung Cancer Registry, Soh et al. found higher local recurrence rates in clinical stage I NSCLC after segmentectomy (64.4%) than lobectomy (52%) (*p* = 0.023). In cT1c patients, segmentectomy was associated with worse outcome especially for tumors with a CTR of 0.5-1, showing significantly worse 5-years OS (*p* = 0.001) and DFS (*p* = 0.024) [8].

In the JCOG0802/WJOG4607L trial, the 5- years relapse free survival was similar between segmentectomy and lobectomy groups (88% vs.87%, *p* = 0.9889, but it identified a higher risk of locoregional recurrence in the segmentectomy group (11% vs. 5% *p* = 0.0018), which makes local control in sublobar resections an important issue, even for solid tumors that are smaller than 2 cm in size [5, 17].

Numerous studies have identified the key risk factors for recurrence in NSCLC emphasizing the importance of complete macroscopic and microscopic resection, as well as maintaining sufficient surgical margins [7, 18, 19]. In our study, R1-resection was only seen in the segmentectomy group. In addition, resection margin was significantly smaller in the segmentectomy group with a significantly smaller proportion of MTR ≥ 1 in this group. This suggests that, anatomically, performing a segmentectomy can make it challenging to achieve sufficient surgical margins especially for larger tumors. This highlights the importance of using preoperative 3D-reconstruction to plan the appropriate type of resection (segmentectomy vs. lobectomy) based on the simulated resection margin.

Recurrence rates differ in literature. Kamigaichi et al. found comparable recurrence rates between segmentectomy and lobectomy for radiologically pure solid IA3 NSCLC (*p* = 0.88) [9]. Interestingly, no local recurrence (defined as tumor recurrence in the preserved lobe) was reported in the segmentectomy group. Chan et al. reported similar results showing no significant difference in recurrence free survival in their study [10]. However, results of these studies cannot be directly compared to ours since their cohorts included patients with pathologic tumor and nodal upstaging, as well as patients who underwent adjuvant chemotherapy.

Hattori et al. also reported no significant difference in the outcome of segmentectomy and lobectomy for clinical stage IA3 NSCLC, but in his analysis he excluded pure solid tumors [11]. The relevance of CTR was further supported by the JCOG1211, a multicenter, single-arm phase 3 study, conducted on segmentectomy for ground-glass dominant lung cancer with a diameter up to 3 cm. It showed that for tumors with a size from more than 2 cm to 3 cm and a CTR of 0·50 or less, the 5-year recurrence free survival after segmentectomy was 98.0% [7]. This suggests that segmentectomy is a suitable curative treatment in this case. Tumors that are predominantly ground glass opacity are less invasive and local control of the main tumor is typically sufficient to treat them [20]. Even the presence of a minor ground glass opacity component in NSCLC has been associated with a better prognosis, while pure solid NSCLC is known for its highly aggressive and malignant characteristics [21, 22]. In our study, almost all patients had pure solid NSCLC, this likely contributed to the worse DFS observed compared to JCOG1211.

In our study there was no difference in overall survival between the segmentectomy and lobectomy group. This has also been found in most other studies [9–11].This might be attributed to the fact that segmentectomy preserved cardiopulmonary reserve, which makes further treatment and even resection in case of local relapse possible. Conversely, Cao et al. found a significant difference in overall survival, with segmentectomy associated with worse outcomes for tumors measuring 2.1 to 3.0 cm compared to lobectomy [23] Our study has several limitations inherent to its retrospective design and single-center analysis. Indications for segmentectomy are not consistently available. Even though there was no difference in the distribution of main clinical parameters between both groups, it is possible that unmeasured factors may explain the difference in outcome. The patient cohort was heterogeneous regarding the type and numbers of segments resected, including single segmentectomies and bisegmentectomies in the segmentectomy group. The use of preoperative assessment of resection margin was not exclusively based on 3D reconstruction software. As we have analyzed patients with pathological stage IA3, no information on upstaging can be given. However, we did find that the number of dissected lymph nodes was significantly lower in the segmentectomy group. Therefore, it might be possible that positive lymph nodes might have been missed in some patients, explaining the rate of locoregional relapse of about 7% in the segmentectomy group.

## Conclusion

Segmentectomy is associated with significantly worse disease-free survival compared to lobectomy for patients with stage IA3 NSCLC. OS was not different between both groups. Further large prospective studies are necessary to give treatment recommendations regarding the indication for segmentectomy in patients with stage IA3 NSCLC.

## Data Availability

No datasets were generated or analysed during the current study.

## Abbreviations

SD: standard deviation
ECOG: Eastern Cooperative Oncology Group Performance Status scale
FEV1: forced expiratory volume in 1 s
DLCO: diffusion capacity of lungs for carbon monoxide
CTR: Consolidation to tumor ratio
